# Genomic Analysis of Purebred and Crossbred Angus Cows Quantifies Heterozygosity, Breed, and Additive Effects on Components of Reproduction

**DOI:** 10.3390/ani12010061

**Published:** 2021-12-28

**Authors:** Wayne S. Pitchford, Judith M. Pitchford, Jena G. Alexopoulos, Michelle L. Hebart

**Affiliations:** Davies Livestock Research Centre, School of Animal and Veterinary Sciences, University of Adelaide, Roseworthy, SA 5371, Australia; judith.pitchford@adelaide.edu.au (J.M.P.); jena.alexopoulos@adelaide.edu.au (J.G.A.); michelle.hebart@adelaide.edu.au (M.L.H.)

**Keywords:** beef, crossbreeding, maternal, genomics

## Abstract

**Simple Summary:**

This study reports a genomic analysis of purebred Angus and Hereford × Angus maternal productivity, which is a key driver of sustainable systems. Heterozygosity effects quantify hybrid vigour or heterosis and were significant for growth and puberty. Breed differences were mostly due to heterosis, but there was an advantage of Hereford genes for reproductive performance. Days to calving was the most important Estimated Breeding Value (EBV) as a predictor of attainment of puberty and subsequent reproductive performance. Genomics offers a cheaper and faster strategy for the development of multi-breed EBVs in commercial herds.

**Abstract:**

Multiple studies have quantified the production differences of Hereford Angus crossbreds compared to purebred Angus for a range of traits including growth, carcass, and reproductive traits. This study aims to quantify breed and heterosis effects on maternal performance using genomics. Thirty Hereford and thirty Angus sires were mated to 1100 Angus heifers and cows in a large commercial herd run on pasture at Musselroe Bay, Tasmania, Australia. Approximately 1650 calves were born. Heifers were weaned, scanned for attainment of puberty prior to joining at approximately 15 months of age, joined, and then recorded for status of pregnancy, calving, lactating, 2nd pregnancy, and weaning of second calf. Heterozygosity effects were significant for heifer pre-joining weight and height as well as proportion pubertal. Breed differences were significant for the same traits plus pregnancy rate at second joining and proportion rearing two calves. Genetic parameters were reported for 13 traits. On average, higher genetic merit (Estimated Breeding Value, EBV percentile) Hereford bulls were used than Angus for growth and puberty, but they were similar for fat and reproduction. Days to calving BREEDPLAN EBVs of the sires were related to puberty and reproduction. Scrotal size BREEDPLAN EBVs of the sires were related to attainment of puberty genomic EBVs calculated. In summary, breed differences in growth and puberty were due to heterosis, but there was an advantage of Hereford genes for reproductive performance. Ongoing emphasis on selection for reduced days to calving and estimation of multi-breed EBVs is important.

## 1. Introduction

Breeder herd or maternal productivity is important for the sustainability of beef cattle industries worldwide because of the large proportion of feed across the production system required for cow maintenance. Indeed, achieving a calving interval of 365 days represents the single most important production issue affecting maternal productivity [1,2]. In Australia, a large trial was conducted following concerns from beef cattle breeders that significant genetic improvement in feedlot and abattoir performance of cattle could have led to a decline in maternal productivity, especially manifest as low reproductive rates during poor pasture growth seasons. The concern was addressed using both a designed experiment on research stations and collecting additional measurements on performance-recording seedstock herds [3]. Leading seedstock producers were interviewed, and it was evident that there are quite different approaches to running cows to maximize sustainable maternal productivity, even between very similar farms [4].

In the research herds, the genetically High-Fat heifers (higher Rib Fat EBV) had higher conception rates than Low-Fat, especially under a shorter joining period [5]. At second joining (maiden lactation), while the Low-Fat line had slightly lower pregnancy rates and were leaner, the differences from High-Fat were small [6]. It could be concluded that this means that the original industry issue is smaller than originally proposed, but equally that the cows were not managed under the conditions observed in commercial production. There were negligible differences between lines in reproductive performance of mature cows [7], but Low-Fat cows continued to be leaner than High-Fat cows and were always those that triggered the initiation of supplementary feeding [8]. Due to the higher reproductive rates in younger cattle, the High-Fat line was more efficient despite eating more than the Low-Fat line [1]. However, economic modelling of a self-replacing herd concluded the reverse for gross margin [9].

Limitations of the Beef Cooperative Research Centre (CRC) maternal productivity trials [3] were that stocking rates were not as high as some in commercial practice, pubertal measures were not included to understand drivers of early pregnancy rates, and there was no effort to quantify breed and heterosis effects. There are many trials in Australia, the USA, and other countries that have reported advantages of “Black Baldy” cattle [10,11,12,13,14,15,16]. These are most commonly Hereford × Angus crosses, inheriting the white face of the Hereford and black coat colour of the Angus. When breeds are crossed there is a gain from both hybrid vigour (heterosis) and from the genes contributed by each breed. More than forty years ago, Cundiff et al. [10,11] reported heterosis effects averaged across Hereford, Angus, and Shorthorn crossbred compared to purebred cows [10,11,12]. They reported higher birth and weaning weights for the crossbreds.

Since that time, there have been ongoing reports of the advantages of crossbred cows. More recently, Daley and Earley [14] reported that Hereford × Angus crosses had advantages in pre-weaning growth and pregnancy rates of heifers, but carcass quality favoured the pure Angus. Similar projects have been conducted by Circle A Ranch (Iberia, MO, USA) and Simplot Livestock Co. (Grand View, ID, USA) [15,16], with the Circle A Ranch trial supporting greater pregnancy rates of Hereford cross than purebred Angus cattle. However, all trials did not have all four breed combinations and so were unable to separate heterosis and breed effects. Similarly, the Australian “Black Baldy” trial also only included Angus cows [17,18]. The Australian trial was designed to have multiple sires per breed (approximately 30) with sires chosen from those being used in industry benchmarking programs [17,18].

The purpose of this paper is to report genetic effects on the components of female reproduction in the Australian Black Baldy trial. The first aim is to quantify the heterosis (or at least heterozygosity) effects separately from the breed effects. Second, to report genomic parameters for the traits. The third aim is to report the relationship between current BREEDPLAN EBVs [19] on the sires and the reproductive performance of their daughters in a commercial herd.

## 2. Materials and Methods

The Black Baldy trial was conducted over five years, 2014 to 2019 [17]. The heifers and steers in the project were born and raised by Musselroe Beef (Launceston, Tasmania Australia) on a property located near Cape Portland in the northeast of Tasmania (latitude 41, longitude 148). This property has a coastal Mediterranean environment with sandy soils and an average rainfall of 677 mm. The 1100 Angus dams used in the trial were in different management groups defined by the standard letter for year of birth of the dam (H 2012, J 2013, K 2014, or L 2015 drop). Eight calf management groups were defined as a function of cow group and birth year with two cow groups calving in 2015 (H, J) and three in 2016 (H, J, K) and 2017 (J, K, L). The 2017 L drop dams were project heifers born in 2015. The dams and their progeny were raised under commercial conditions on pasture with no supplementary feeding.

Over three years (2014, 2015, and 2016), Angus heifers (2 years old at calving) and cows (3 or 4 years old at calving) were mated to leading Angus or Hereford bulls by artificial insemination to produce the heifers of interest. The sires included bulls that were used in benchmarking programmes or were widely used in their respective breeds [17]. Angus bulls were used as back-up bulls. The exception to this were the L heifers (Angus or Hereford sired) in Cohort 3 that were naturally mated to either Angus or Hereford bulls and have their performance reported herein. The Angus dams were commercial cattle, and most were purebred, but some were 50% Hereford and some 25% Hereford. Thus, the Angus sired calves were 75–100% Angus and the Hereford sired calves were 50–75% Hereford.

Calving occurred in winter/spring. Cohort 1 calved in June–August 2015, Cohort 2 in June–August 2016, and Cohort 3 in July–September 2017. Approximately 1100 cows were mated, and many were mated over multiple years. There were 1562 calves (male and female) sampled at weaning in February–March and subsequently genotyped. The 755 (Table 1) heifers were raised to late September when they had weight, height, and P8 rump fat depth measured, and they were ovarian scanned to determine if they were pubertal. Two previous scans for puberty were conducted in June and August but not reported. Then, heifers deemed of sufficient size by the veterinarian (684 of 725 yarded pre-joining) were naturally joined to Hereford and Angus bulls, pregnancy tested in summer, calved down the following winter, joined again, pregnancy tested again, and then calves were weaned in February–March. Project funding ceased, and so the level of recording for Cohorts 2 and 3 was less than that for Cohort 1. The last calves (second calf of 2017 born cows) were weaned in March 2021.

Not all heifers completed the two reproductive cycles, with culls at each stage. The number that were scanned for puberty is a proportion of the number at weaning (Table 1). The number of heifers in the first joining is given as a proportion of number of heifers that were scanned. Likewise, the number of heifers that became pregnant is given as a proportion of the number that were joined, and the number of heifers that calved is given as a proportion of the number that were pregnant. This is the same for the second calving. Overall, the heifers that raised two calves is given as a proportion of the number of heifers at the first joining.

Genomic data: Cattle were genotyped on multiple platforms with 12 animals on GGP100K, 25 on GGPHD140K, 352 on GGPLD30K, and 1106 GGP50K chips. Most but not all sires were genotyped. There were ample purebred Angus genotypes, but 1398 purebred Hereford genotypes (GGP50K) from outside this study were added to assist with the imputation of Hereford haplotypes to a common chip (GGP50K) prior to genotype cleaning and genomic relationship matrix (GRM) construction. The 37 sires with >100K single nucleotide polymorhpisms (SNPs) were cut down to 50K and included in the reference population. There were 12,340 SNPs in common between the GGPLD30K and GGP50K chips. Imputation was performed using Fimpute 3 [20]. SNPs were removed from the reference genotypes if they were unable to be mapped to precise chromosome/base pair location or mapped to the X or Y chromosomes. SNPs with identical chromosome or base pair positions were merged. The result was a target set of 42,205 SNPs. After imputation, SNPs were excluded that had a minor allele frequency (MAF) less than 0.01 across both pure and crossbreds for a final SNP count of 40,743 to be used for GRM construction.

Homozygous genotypes for the major allele were coded as 0, those for the minor allele were coded as 2, and heterozygous genotypes were coded as 1. The GRM was constructed as per VanRaden’s first method [21];

$$\frac{ZZ^{\prime }}{2\sum_{i=1}^{n}p_{i}\;\left(1-p_{i}\right)}$$

where ***Z*** denotes a centred matrix of allele effects with a mean of zero of size number of animals by number of markers, ***Z’*** is the transpose of ***Z***, *n* is number of animals, *p_i_* is the frequency of the minor allele at locus i, and division by $2\sum p_{i}\;\left(1-p_{i}\;\right)$ scales the **G** matrix to be similar in magnitude (so that diagonal elements average 1) to the numerator relationship matrix constructed from genealogy [21].

Only those animals genotyped are included in the analyses, so the numbers differ slightly from those of Pitchford et al. [17]. There were 1562 genotyped progeny from 30 (28 with BREEDPLAN EBVs) Hereford sires and 22 (21 with BREEDPLAN EBVs) Angus sires. Some of the Angus back-up sires used early in the project were not genotyped.

Statistical analysis: To account for variation between cows of different age and for different seasons between calving years, a management group combining cow age and calving year was created with eight levels (H2015, H2016, J2015, J2016, J2017, K2016, K2017, L2017).

As mainly Angus bulls were used as back-up bulls, there were more Angus sired calves born late, and so, they tended to be younger than Hereford sired calves. To account for this, a Calving Time factor was developed based on ovarian cycle length where calves were born early (26 days from first calf born), medium (next 21 days), or late (more than 47 days from first calf born). The contemporary group was defined as Calving Time factor nested within the management group. Day of birth was fitted as a linear covariate within contemporary group. Of 1562 calves at weaning, 652 were sired by Hereford and 910 by Angus bulls.

Data were analysed with a linear mixed model using ASReml-R version 4 [22]. Fixed effects for all traits included contemporary group and Date of Birth nested within it. Two models were used to analyse the data: (1) Breed differences were analysed by fitting the contemporary group with day of birth plus sire breed as fixed effects and sire as a random effect; and (2) Univariate genetic effects were modelled by fitting the contemporary group with day of birth plus a fixed covariate heterozygosity based on the proportion (%) of markers that were heterozygous and a random genomic relationship matrix. Binary traits were treated as continuous to simplify calculations and bivariate model fitting. Bivariate models had the same effects fitted as the univariate genetic models with genetic and environmental covariances estimated.

## 3. Results

### 3.1. Breed Differences and Heterosis

Hereford sired heifers were 5.6% heavier and 1.4% taller pre-joining (Table 1). There were also 12% (31% vs. 19%) more that were pubertal pre-joining, although many of those not pubertal pre-joining still conceived. Breed differences were not significant for most components of reproductive performance. However, there were 12% (49% vs. 37%) more Hereford-sired than Angus-sired heifers that were joined and proceeded to successfully raise two calves. This occurred despite 6% (14% vs. 8%) greater calf death rates from maiden Herefords.

The mean heterozygosity for Angus and Hereford sired calves was 41.1% and 44.6%, respectively. Thus, the difference of 3.5% can be multiplied by the heterozygosity value (Table 1) to obtain an estimate for heterosis. When this was done, the estimated heterosis effect for pre-joining weight was 3.6%, height 0.9%, P8 fat depth 6.1%, and proportion pubertal 13%. This clearly demonstrates that the majority of the additional growth and proportion pubertal of the Hereford sired calves is due to heterosis rather than breed differences. On average, the Hereford and Angus sires used in the project were close to breed average, and there were similar percentiles for most traits (Table 2) with the exception that the Hereford bulls were better for growth (top 20% vs. 60%) and scrotal size (top 20% vs. 45%). It is likely that the difference in genetic merit of bulls within the breeds also accounted for some of the breed differences in early traits.

The heterozygosity effect on proportion of cows weaning two calves was not significant, and when calculated as a heterosis effect, it would only account for a difference of 5%, which is less than the 12% difference between the sire breeds (Table 1). Thus, there appears to be an additive genetic difference (Hereford advantage) between the sire breeds. Given that on average, sires from both breeds were close to the breed average or slightly above for days to calving (Table 2), it is likely the Hereford advantage in reproductive rate is real.

### 3.2. Heritability and Genetic Correlations

Pre-joining (yearling) weight (37%) and height (39%) were moderate to highly heritable, as expected. P8 fat depth was less so (22%) but likely as expected given that the mean fat depth was only 2 mm (Table 1). The heritability of components of maternal productivity is often low, especially when binary traits are close to zero or one, so there is little variation. The heritability of proportion pubertal and pregnant both had significant variation and heritabilities over 10%, demonstrating both significant genetic and residual variation (Table 1). The heritability of two weaned calves was 8% with breeding values of sires ranging from −17 to +11% and genetic standard deviation of 13%, which demonstrates significant opportunity for genetic improvement.

Genetic and phenotypic correlations have been estimated (Table 3). The genetic correlation between pre-joining weight and height was high (0.78). Height was not correlated with most other traits, but weight was correlated with P8 fat depth (0.39), proportion pubertal (0.36), proportion pregnant at second joining (0.34), and proportion of cows rearing two calves (0.65). The genetic correlation between pregnancy outcomes at first and second joining was high (0.73). Somewhat surprisingly, the correlation between attainment of puberty pre-joining and pregnancy rate was higher for the second joining (0.67) than the first (0.39). The correlation between P8 fat depth and proportion rearing two calves was extremely high (0.96). Two genetic covariances with proportion weaning two calves were non-estimable, which was possibly because they were close to unity. Thus, in addition to the bivariate models, the GEBVs from univariate models were correlated as an indication of genetic correlations. The correlation between weaned two calves and PTIC2 was greatest (0.84); then, it was moderate for puberty (0.41) and PTIC1 (0.43), and it was quite low for weight (0.30), height (0.18), and P8 fat (0.18). This suggests that the extremely high 0.96 correlation between P8 fat and weaned two calves was not real.

Phenotypically, the pre-joining measures of weight, height, fat, and puberty were lowly correlated with proportion rearing two calves (0.10–0.16). Pregnancy outcomes at first (0.46) and second (0.81) joining were highly phenotypically correlated with proportion rearing two calves. However, this was largely causal, as they are components of the trait that had the highest variation.

### 3.3. BREEDPLAN EBVs

The purpose of the analysis was not to benchmark the sire breed performance relative to the national system (BREEDPLAN) as described by Pitchford et al. [18], because different traits were measured. However, there is the potential to test the impact of current EBVs on components of maternal productivity in a commercial herd. Genomic breeding values of sires used for the traits recorded were regressed on their BREEDPLAN EBVs for a range of traits expected to be related (Table 4). A separate intercept for the two breeds was included in the model to allow for the different base for each breed.

Weaning weight was used rather than yearling weight, as it is separated into both direct and maternal components and provided the hypothesis of whether milk production was associated with the pregnancy rate of lactating cows. While there were only weak relationships, the regression of second pregnancy rate on milk EBV was significant (*p* < 0.10) with a slope of 0.37% lower pregnancy rate per 1 kg maternal EBV. If the genetic standard deviation (SD) for milk EBV based on percentile tables is 2.8 kg, then this relationship represents an approximately 1% pregnancy rate per milk EBV SD and so is not a large effect. The first pregnancy rate was also significant but a 0.27% increase per kg. The 200 day direct or growth EBV was associated with (*p* < 0.10) a 0.23% lower proportion pubertal per kg. The sire P8 fat EBV was not related to any of the traits despite the same trait being measured. It is assumed this is due to the lower mean (2.35 mm) and variation (σ_P_ = 0.81) than would commonly be the case.

As expected, the days to calving and scrotal size EBVs were both related to proportion pubertal. Calculated from percentile tables, if the standard deviation in days to calving EBV is approximately 1.25 days and scrotal size EBV is 0.6 cm, then the increase in proportion pubertal (Table 4) represents 1.2% per SD in days to calving and 1.8% per SD in scrotal size. Similarly, the effect on proportion weaning two calves was approximately 1.0% per SD in days to calving and 0.6% per SD in scrotal size.

## 4. Discussion

### 4.1. Breed Differences and Heterosis

This project was initiated by Hereford breeders wanting to demonstrate the value of using Hereford sires in commercial Angus herds. Pitchford et al. [17,18] reported that, as expected, there was some gain in growth from Hereford sire calves but with limited change in carcass value owing to the lower marbling of Hereford sire calves (depending on premiums paid for quality). Furthermore, it has been demonstrated that much of the benefit in growth is due to heterosis rather than breed differences [18] (Table 1). Daley and Earley [14] reported similar results for steers, and in addition, there was a 7% higher pregnancy rates in Hereford than Angus-sired heifers.

There are multiple studies that quantify the effect of heterosis on reproductive performance. Cundiff et al. [10,11] reported a heterosis effect across multiple breed crosses of 6.4% more calves weaned, and this was due to increased conception rates. Pitchford et al. [23] reported heterosis effects in Brahman × Hereford crosses that were largest for growth on high-quality pasture but largest for reproduction on low and medium-quality pasture (over 0.9 more calves per three opportunities vs. 0.36 on high quality pasture). The cattle in this trial were on high quality pasture but at high stocking rates. Thus, there was likely ample opportunity to express heterosis for reproductive performance given that pregnancy rates were significantly less than 100%. The 4.6% more cows weaning two calves is lower than reported by Pitchford et al. [23] but similar to comparable breed crosses reported by Cundiff et al. [10,11].

### 4.2. Heritability and Genetic Correlations

Johnston and Bunter [24] modelled days to calving data in Angus cattle and covariances with calving success. The variations in calving success (σ_P_ = 0.37) and heritability (0.11) were similar (Table 1). They found the genetic correlation with days to calving was −0.97, so the genetic regression coefficient would be 1.5% more calves per day reduction (improvement) days to calving compared to values just under 1% (Table 4).

Meyer and Johnston [25] reported genetic correlations between days to calving and ultrasound scan traits in Hereford cattle. The heritability of days to calving was only 3%, but the genetic correlation with P8 fat depth in heifers was quite strong −0.65. This is similar to the P8 and pregnancy rate correlations for first (0.46) and second (0.82) joining (Table 3). With more data, it is unlikely that the relationship between fat and weaning two calves is as strong as implied by the 0.96, but given reports of quite strong relationships with days to calving and in high stocking rate production systems, it could genuinely be high despite the low correlation between univariate GEBVs calculated (Table 3). Weik et al. [26] recently published genetic parameters for similar traits. The heritability of rebreeding (Pregnant 2) was 0.13, which is similar to 0.19 (Table 1). Their genetic correlation with heifer weight was similar (0.19 vs. 0.34, Table 3), but with height, it was quite different (0.54 vs. −0.04).

As covariances between weaned two calves and some component traits were difficult to estimate (Table 3), the correlations between genomic breeding values from univariate analyses have been included. Correlations with weight (0.30), height (0.18), and P8 fat depth (0.18) are lower and, certainly for fat depth, much more realistic than the 0.96 estimated from the bivariate analysis. Furthermore, correlations with the component traits are all estimable. Breeding values for weaned two calves were correlated with attainment of puberty (0.41) as well as first (0.43) and second (0.84) pregnancy test. Given that these are components of the composite trait, it is absolutely expected that they would be moderate–highly correlated.

The regression of sire GEBV on BREEDPLAN EBV for scrotal size was only significant for attainment of puberty. Morris et al. [27] published parameters from Angus selection lines and reported genetic correlations between bull scrotal size and heifer age at puberty (−0.25) as well as first (0.14) and second (0.25) pregnancy rate. The associated genetic regression coefficients calculated from their paper are 1.3% and 1.7% higher pregnancy rate per 1 cm scrotal size for first and second joining, respectively. These values are similar to the values of 1.0 and 0.7% for first and second, respectively (Table 4). Johnston et al. [28] reported genetic correlations between male and female reproduction traits in Brahman and Tropical Composite cattle. The correlation between yearling scrotal size and pubertal prior to joining in Composites was 0.15, age at puberty −0.21, and weight at puberty 0.23. The correlation between Composite heifer pubertal pre-joining and pregnancy rate at first and second joining was 0.68 and 0.47, respectively, which aligned to 0.39 and 0.71 (Table 3). The finding that pregnancy rate was most closely associated with the days to calving EBV was both expected and matches exactly the conclusion of Jones et al. [5].

## 5. Conclusions

Maternal productivity is key driver of sustainability due to the large feed requirement for the maintenance of breeder herds and also the importance of weaning rate during periods of herd rebuilding after liquidation during drought. This project has quantified the differences in components of maternal productivity when using Hereford compared with Angus sires in an Angus cow herd. Using genomics allowed the separation of heterosis and additive genetic effects, thus enabling quantification of the effect of current BREEDPLAN EBVs on maternal traits. As expected, the days to calving EBV was the most related to maternal performance, and the scrotal size EBV was the most related to attainment of puberty in heifers. There was an advantage of using Hereford sires on maternal performance beyond that accounted for by heterosis. Breeders are encouraged to utilise breed differences as well as selecting superior sires within breeds especially focusing on days to calving for maternal performance.

## Figures and Tables

**Table 1:** Number of measurements, sire breed means from fixed effects model, heterozygosity, phenotypic standard deviation (σ_P_), and heritability estimates from the genomic model. All traits except weight, height, and fat are binary (0 = no and 1 = yes).

Trait	Number	Angus Sire	Hereford Sire ^A^	Heterozygosity ^B^	σ_P_	Heritability
Pre-joining weight (kg)	717	294.9 ± 15.4	311.4 ± 15.4 ***	3.0 ***	28.0	0.37 ± 0.09
Pre-joining height (cm)	660	118.9 ± 1.9	120.6 ± 1.9 ***	0.3 **	3.6	0.39 ± 0.09
P8 rump fat depth (mm)	702	2.28 ± 0.45	2.43 ± 0.45	0.04 ^†^	0.81	0.22 ± 0.09
Scanned per weaned	755	0.96 ± 0.01	0.95 ± 0.01	0.001	0.20	0
Pubertal per scanned	721	0.19 ± 0.03	0.31 ± 0.03 **	0.038 ***	0.43	0.11 ± 0.07
Joined per scanned	725	0.92 ± 0.01	0.92 ± 0.02	0.000	0.21	0.07 ± 0.08
Pregnant per joined	673	0.75 ± 0.03	0.77 ± 0.03	0.011	0.38	0.12 ± 0.08
Calved per pregnant	556	0.92 ± 0.03	0.91 ± 0.03	0.000	0.30	0
Dead calf per calved	479	0.08 ± 0.03	0.14 ± 0.03 ^†^	0.005	0.28	0.08 ± 0.11
Joined per calved	512	0.93 ± 0.02	0.90 ± 0.02	0.003	0.21	0.11 ± 0.10
Pregnant per joined	486	0.65 ± 0.04	0.81 ± 0.05 **	0.004	0.43	0.19 ± 0.10
Calved per pregnant	407	0.79 ± 0.04	0.84 ± 0.04	0.004	0.37	0.17 ± 0.12
Weaned 2 calves	684	0.37 ± 0.03	0.49 ± 0.04 **	0.013	0.48	0.08 ± 0.07

^A^ Test of significant difference from Angus; ^B^ test of significant difference from zero in units of trait per percent heterozygosity; ^†^ *p* < 0.10, ** *p* < 0.01, *** *p* < 0.001.

**Table 2:** BREEDPLAN EBVs for bulls used relative to breed average.

Breed	200 Day Growth (kg)	200 Day Milk (kg)	P8 Fat (mm)	Days to Calving (Days)	Scrotal Size (cm)
Hereford sires used	37.7	17.7	1.3	−3.0	2.6
Hereford mean	33.0	16.0	0.6	−2.8	2.0
Hereford percentile	20%	35%	25%	45%	20%
Angus sires used	46.0	17.0	0.0	−5.0	2.0
Angus mean	48.0	17.0	−0.4	−4.8	1.9
Angus percentile	60%	50%	35%	45%	45%

**Table 3:** Genetic (below diagonal) and phenotypic correlations between traits from bivariate analyses and correlations between GEBVs for weaned two calves from univariate analyses.

Trait	Wt	Ht	P8	Pub	PTIC1	PTIC2	W2C
Weight (Wt)		0.58	0.29	0.17	0.16	0.07	0.16
Height (Ht)	0.78		0.14	0.06	0.12	0.00	0.12
P8 fat depth (P8)	0.39	0.08		0.21	0.05	0.11	0.10
Pubertal (Pub)	0.36	0.45	0.46		0.05	0.11	0.12
Pregnant 1 (PTIC1)	0.10	−0.17	0.46	0.39		0.42	0.46
Pregnant 2 (PTIC2)	0.34	−0.04	0.82	0.67	0.73		0.81
Weaned 2 calves (W2C)	0.65	0.28	0.96	NE	0.53	NE	
W2C GEBV correlations	0.30	0.18	0.18	0.41	0.43	0.84	

NE non-estimable. SE of genetic correlations range 0.24–0.43, phenotypic correlations 0.03–0.04.

**Table 4:** Regression of sire GEBVs on BREEDPLAN EBVs.

Trait	200 Day Growth (kg)	200 Day Milk (kg)	P8 Fat (mm)	Days to Calving (Days)	Scrotal Size (cm)
Weight (kg)	0.25	0.11	−0.41	−0.38	2.83 ^†^
Height (cm)	0.028	0.041	−0.149	0.034	0.015
P8 fat depth (mm)	−0.002	0.004	0.004	−0.018	0.056 ^†^
Pubertal (binary)	−0.0023 ^†^	0.0008	0.0026	−0.0099 *	0.0303 ***
Pregnant 1 (binary)	−0.0014	0.0027 ^†^	0.0026	−0.0086 ^†^	0.0100
Pregnant 2 (binary)	−0.0020	−0.0037 ^†^	0.0022	−0.0089	0.0072
Weaned 2 calves (binary)	−0.0015	−0.0006	0.0042	−0.0079 *	0.0098

^†^ *p* < 0.10, * *p* < 0.05, *** *p* < 0.001. SE of sire GEBVs for weight range 10–15 kg and W2C 0.11–0.13 calves.

## Data Availability

The data is owned by Herefords Australia Limited and is not publically available.
